# Supplementary material for: A safe insect-based chikungunya fever vaccine affords rapid and durable protection in cynomolgus macaques
Source: NPJ Vaccines. 2024 Dec 19;9:251. doi: 10.1038/s41541-024-01047-z (PMC11659317; doi:10.1038/s41541-024-01047-z)
Supplement: Supplementary file 1 — Supplementary information [file 41541_2024_1047_MOESM1_ESM.doc]

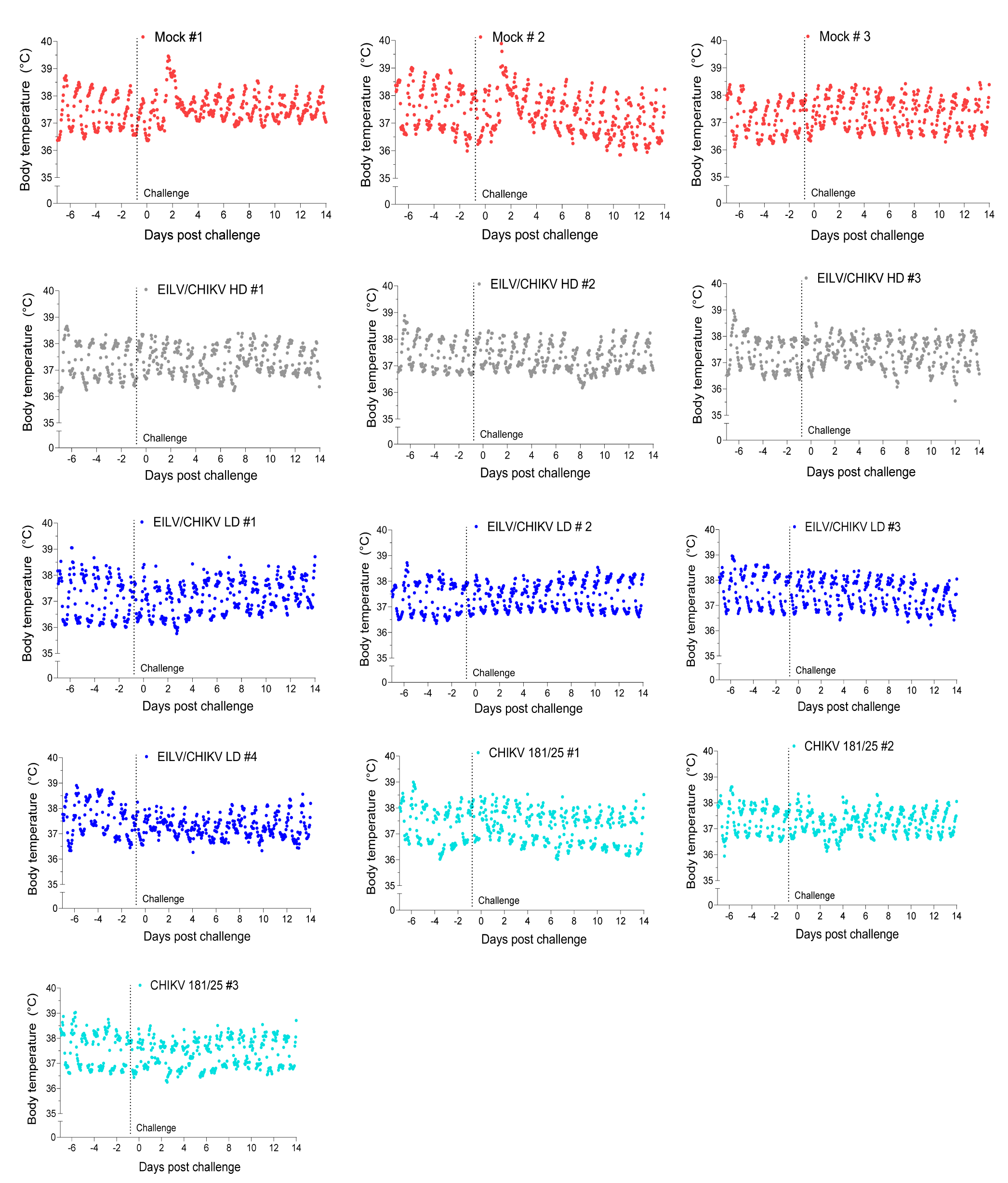


**Supplementary Figure 1. Body temperature changes of CHIKV-infected macaques 1 year post vaccination.** Body temperature changes of individual animal were recorded every 15 min and reported as mean ± standard error of the mean (SEM) starting 7 days before to 14 days after infection with WT CHIKV strain La Réunion.


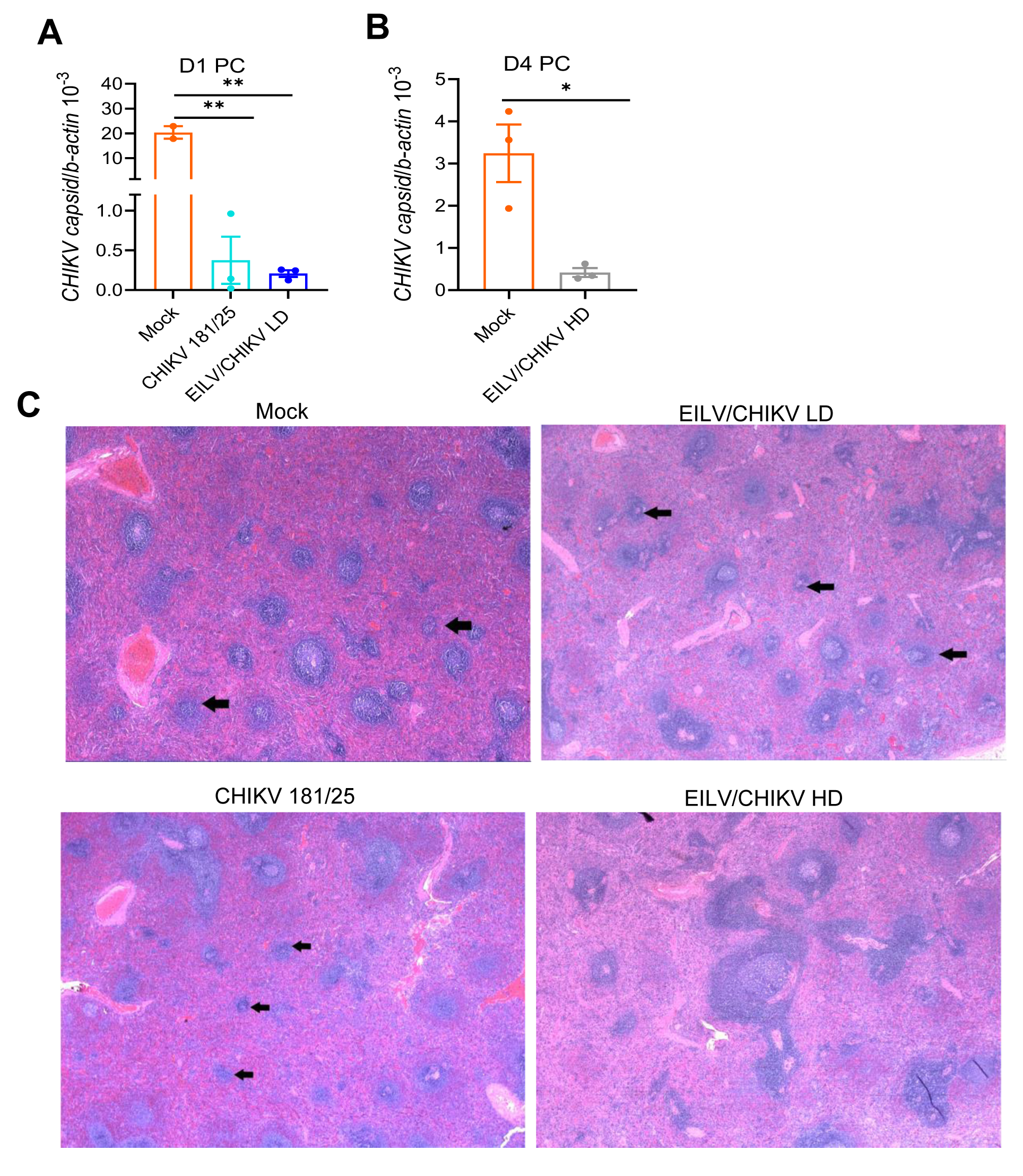


**Supplementary Figure 2. Viremia and histopathology of CHIKV-infected macaques 1 year post vaccination. A-B.** Viremia was measured by Q-PCR at indicated days post-challenge (PC). ** *P* < 0.01 or * *P* < 0.05 compared to mock group. Unpaired, 2-tailed Student’s t test was used to determine the differences. Data are presented as means ± standard error of the mean (SEM). **C.** Representative images of H& E-stained spleens of vaccinated macaques at day 14 PC. Comparatively, minimal to mild decreased lymphocytes in lymphoid nodules (arrows) were observed in some animals.


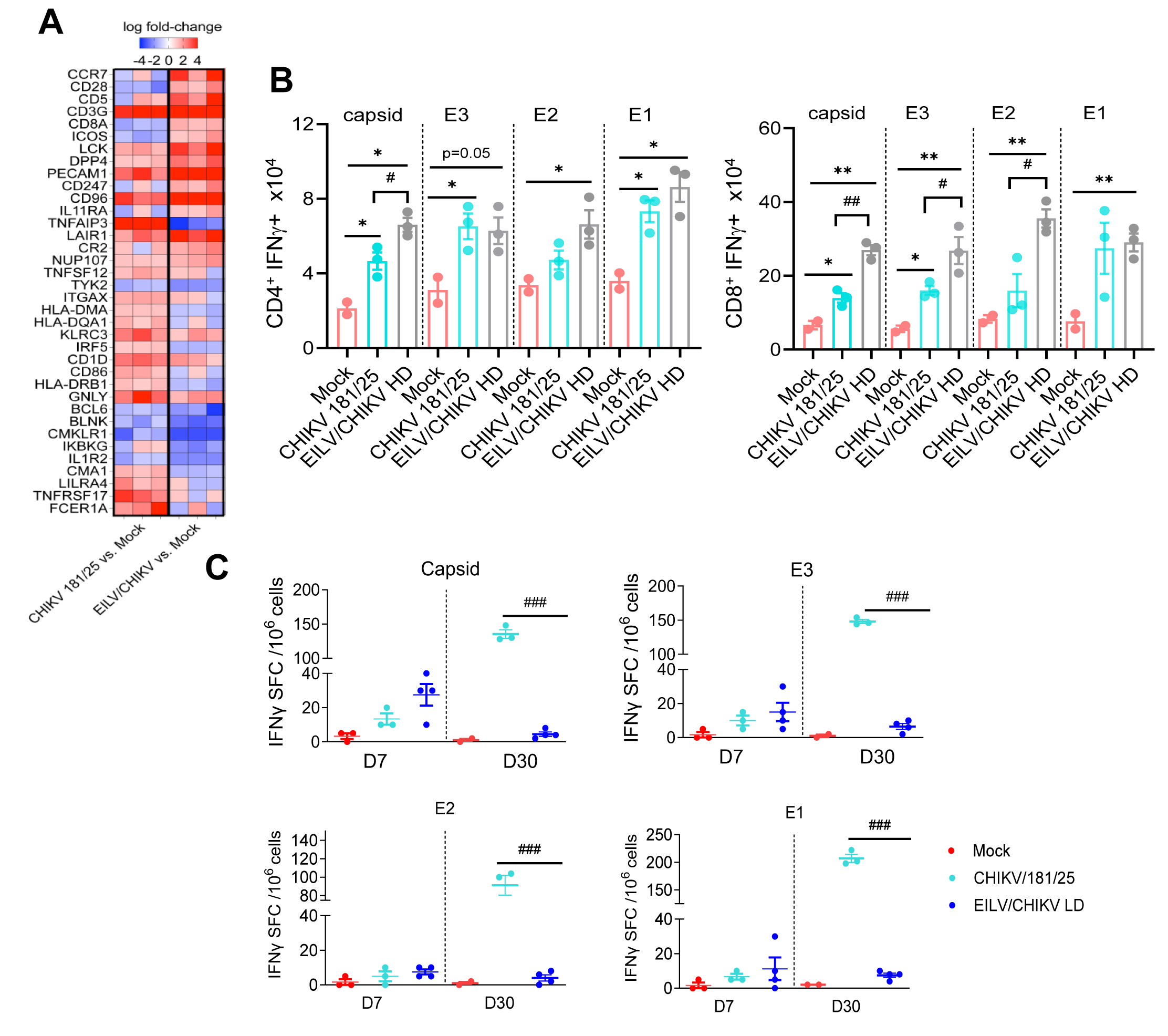


**Supplementary Figure 3. Transcriptional analysis and CHIKV-specific T cell responses in vaccinated macaques. A.** Heatmap of differentially expressed mRNAs in PBMC for individual CHIKV 181/25-vaccinated subjects (n=3) and EILV/CHIKV-vaccinated subjects (n=3) at 4 days post infection compared to mock-immunized subjects; sorted by statistical significance (Benjamini-Hochberg adjusted p-value < 0.05) for the most differentially expressed transcripts between the two vaccinated groups. Red indicates increased expression, white indicates no change in expression, blue indicates decreased expression. **B.** PBMCs of day 30 vaccinated NHPs were cultured *ex vivo* with CHIKV capsid, E3, E2 and E1 peptide pools for 6 h, and stained for IFN-γ, CD3, CD4, or CD8. Total number of IFN-γ^+^ CD4^+^ and CD8^+^ T cells is shown. **C.** ELISPOT quantification of peripheral T cell responses. PBMCs of day 7 and day 30 vaccinated NHPs were stimulated with CHIKV capsid, E3, E2 and E1 peptide pools for 24 h. Spot forming cells (SFC) were measured by IFN-γ ELISPOT. Data are shown as # of SFC per 10^6^ cells. ***P* < 0.01, or **P* < 0.05 compared to mock. ^###^*P* < 0.001, ^##^*P* < 0.01, or ^#^*P* < 0.05 compared to CHIKV 181/25.


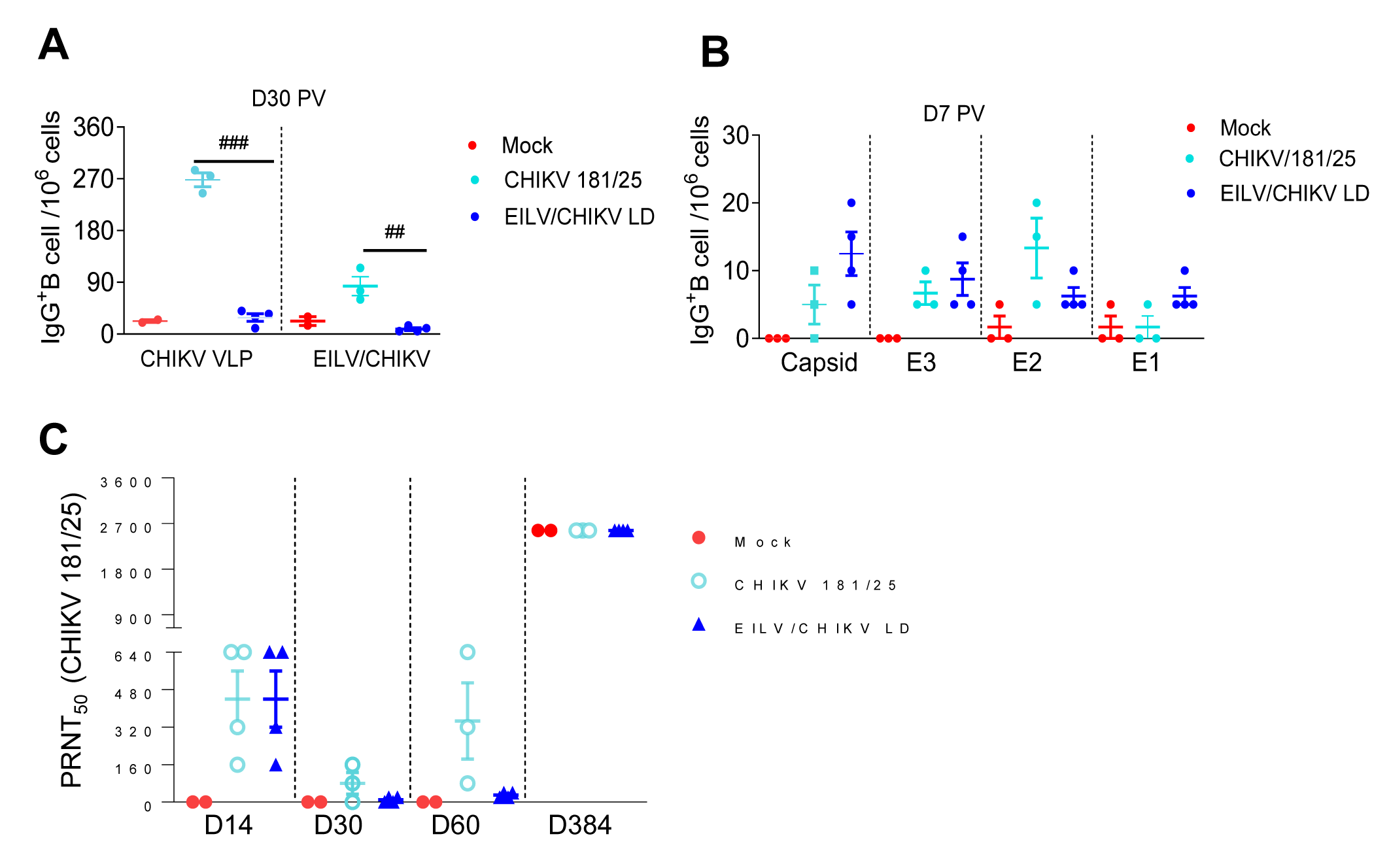


**Supplementary Figure 4. CHIKV specific memory B cell and antibody responses in EILV/CHIKV-vaccinated NHPs in long-term protection study. A-B.** CHIKV-specific memory B cell (MBC) responses by ELISPOT analysis. PBMCs of day 30 (**A**) and day 7 (**B**) vaccinated NHPs were stimulated for 7 d with R848 plus rIL-2 and seeded onto ELISPOT plates coated with (**A**) CHIKV VLP and EILV/CHIKV or (**B**) CHIKV capsid, E3, E2 and E1 peptide pools. The frequencies of CHIKV-specific ASCs per 10^6^ input cells in MBC cultures from the subject. **C.** Neutralizing activity against CHIKV 181/25 in sera collected at various time points PV was measured by PRNT. ^##^*P* < 0.01, or ^#^*P* < 0.05 compared to CHIKV 181/25.


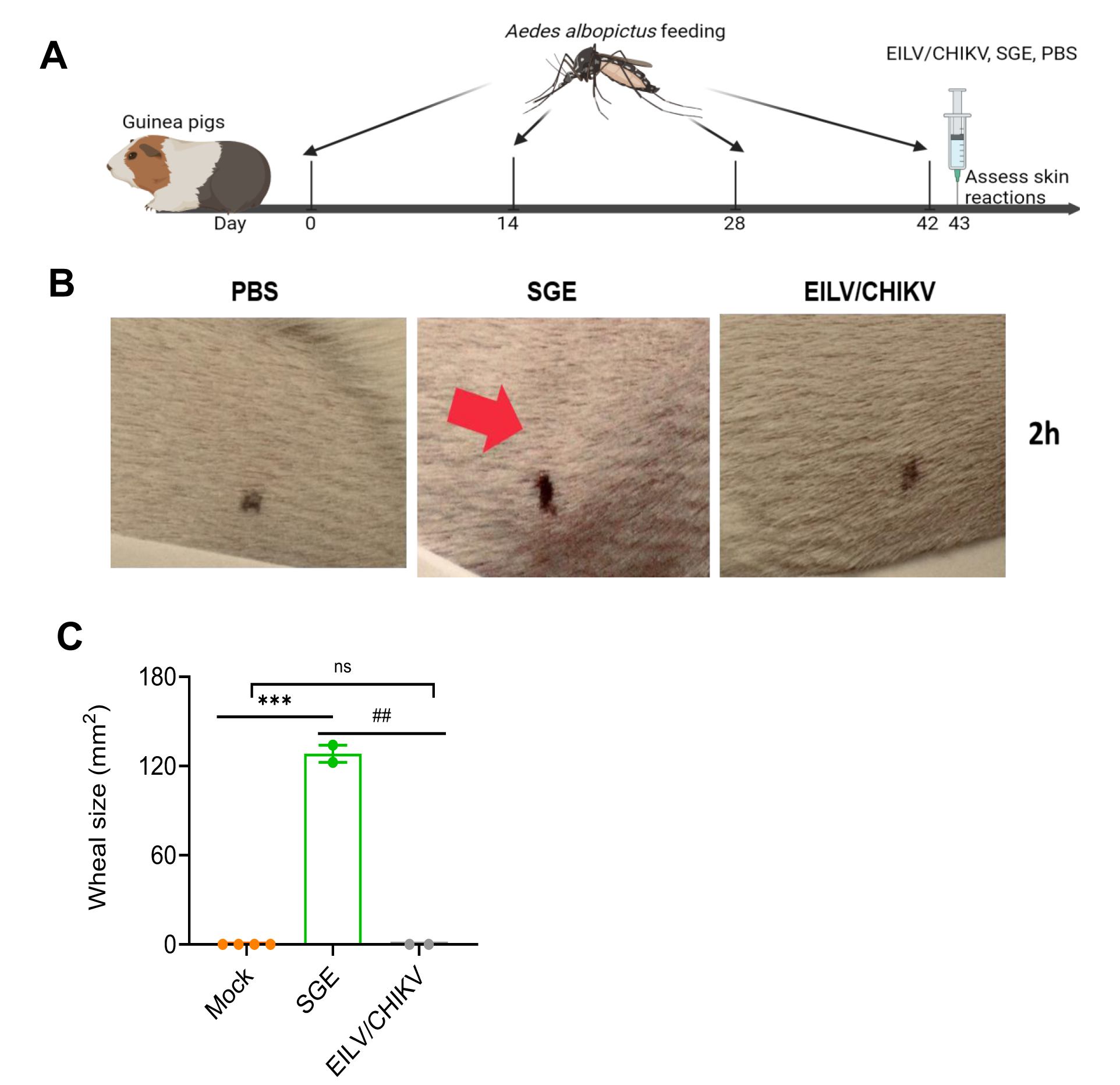


**Supplementary Figure 5. Hypersensitivity assay in a guinea pig model.** Guinea pigs were sensitized by exposure to female *Ae. albopictus* mosquitoes four times during a 14-day period. On day 43, animals were injected i.d. on footpad with 20µg SGE protein, 1.3 x 10^8^ PFU of EILV/CHIKV or PBS (mock). 30 min to 2 h after challenge, skin wheal and flare reactions were assessed. **A.** Study design [Created in BioRender. Wang, T. (2024) BioRender.com/e86u999]. **B.** Representative image of skin wheal and flare reactions at 2 h post injection on day 43. **C.** Skin swelling (wheal) was measured via 30 min post inoculation. ****P* < 0.001 compared to mock. ^##^*P* < 0.01 compared to SGE group.


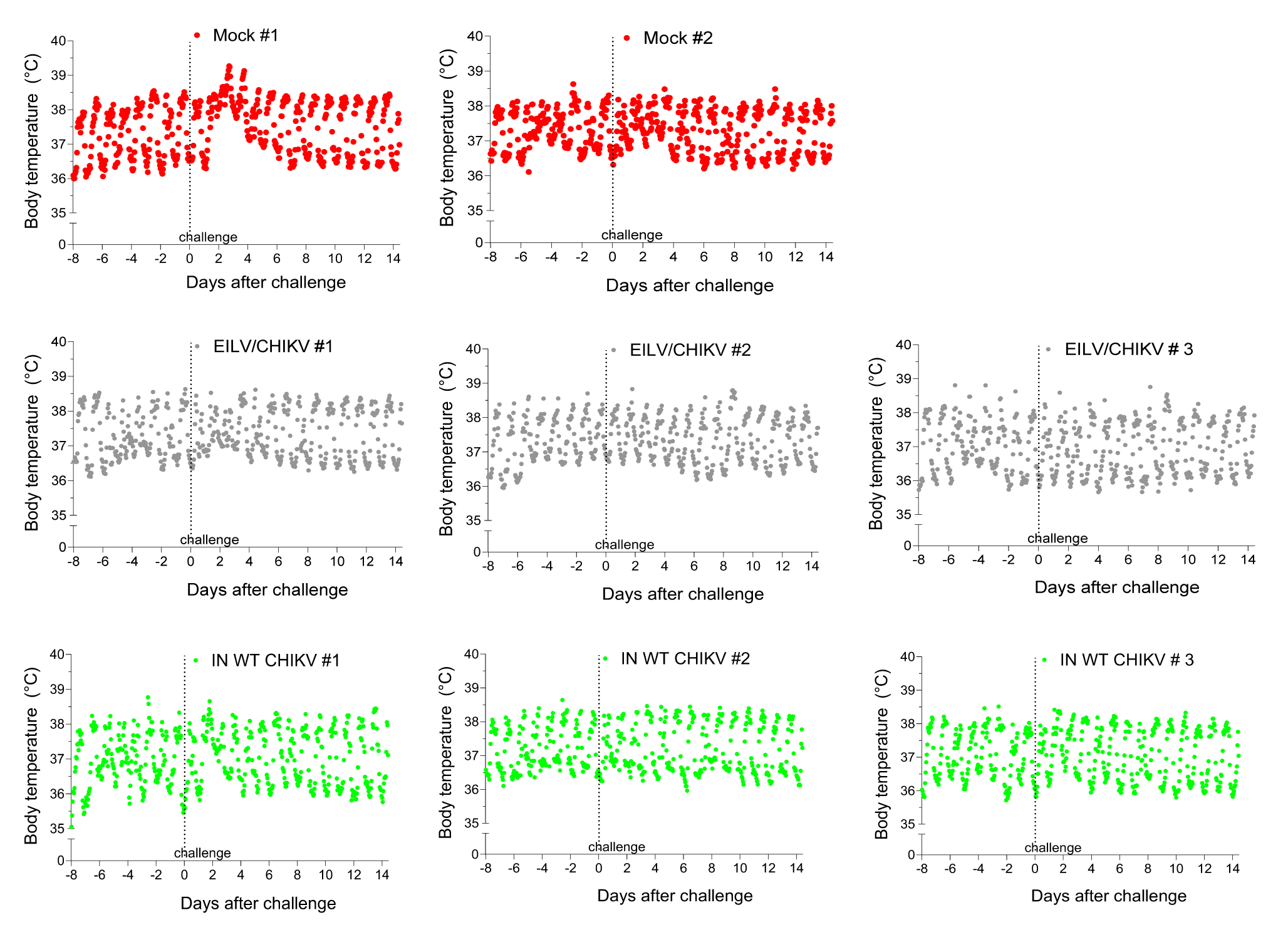


**Supplementary Figure 6. Body temperature changes of CHIKV-infected macaques 6-day post vaccination.** Body temperature changes of individual animal were recorded every 15 min and reported as mean ± standard error of the mean (SEM) starting 7 days before to 14 days after infection with WT CHIKV strain La Réunion.


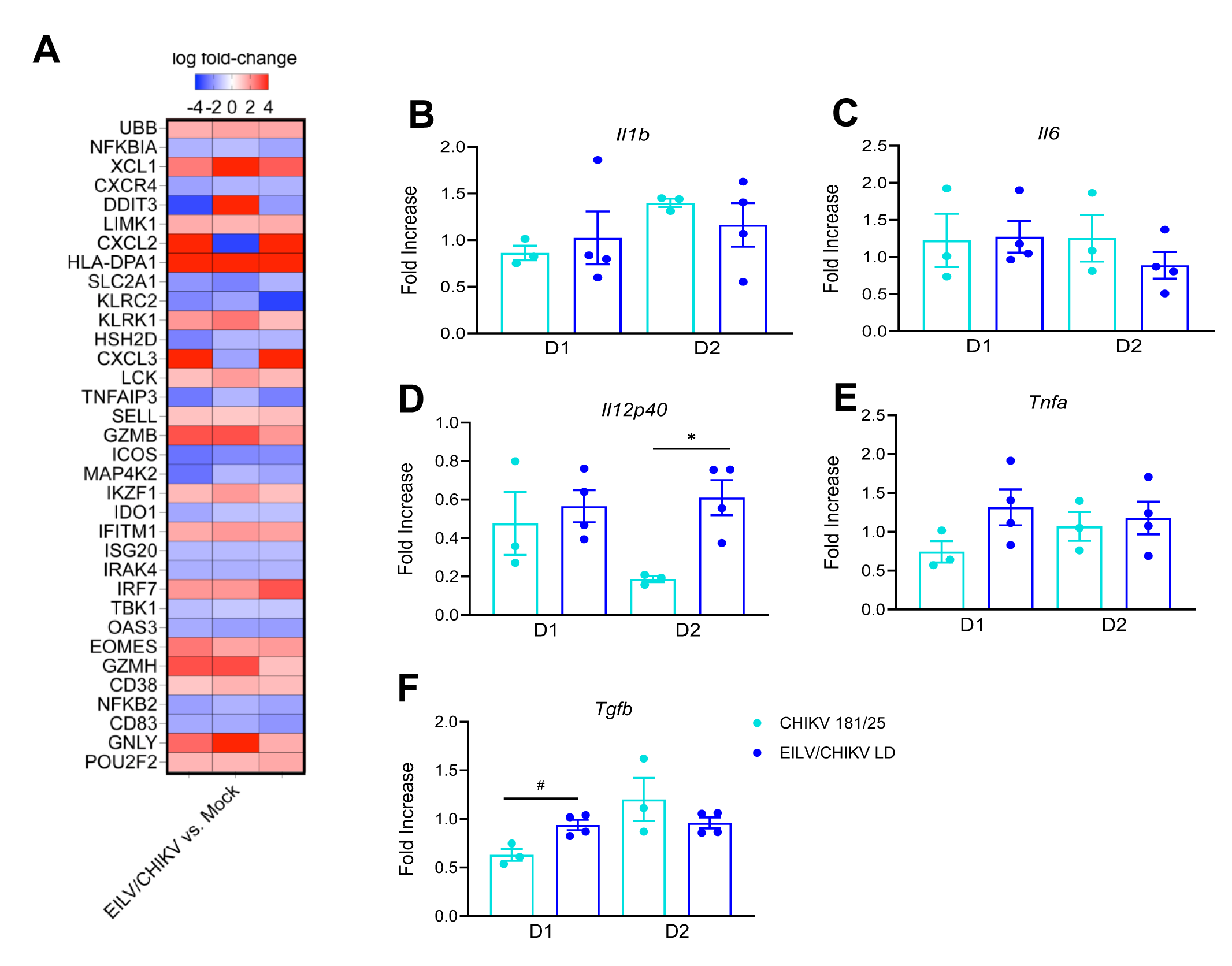
**Supplementary Figure 7. Transcriptional responses in individual macaque subjects and EILV/CHIKV induced innate cytokine responses.** **A.** Heatmap of the most differentially expressed mRNAs in PBMC for individual EILV/CHIKV-vaccinated subjects (n=3) compared to mock-immunized subjects at 7 days post vaccination; sorted by statistical significance (Benjamini-Hochberg adjusted p-value < 0.05). sorted by statistical significance (Benjamini-Hochberg adjusted p-value < 0.05) for the most differentially expressed transcripts between the two vaccinated groups. Red indicates increased expression, white indicates no change in expression, blue indicates decreased expression. **B-F**. NHPs were vaccinated with a LD EILV/CHIKV (n =4), CHIKV 181/25 (n =3) or PBS (mock, n= 3). On days 1, and 2 PV, blood cytokines levels were determined by Q-PCR assay. Data are presented as means ± SEM of fold increase compared to the mock group. ^#^*P* < 0.01 compared to CHIKV 181/25.

**Supplementary Data 1**. Transcriptomic fold-change values for individual macaque subjects immunized with EILV/CHIKV or CHIKV 181/25 at day 7 PV or day 4 PC.

**Supplementary Data 2**. Normalized data (fold change values and Benjamini–Hochberg adjusted p-values) of the transcriptional analysis were exported as an .xlsx file.
